# Exploring Long-Range Surface-Induced Mobility Enhancement in Poly(methyl methacrylate)

**DOI:** 10.1021/acs.macromol.5c01939

**Published:** 2025-09-18

**Authors:** Haoran Nie, Xiwen Chen, Zongyi Ma, Rui Zhang, Ophelia K. C. Tsui

**Affiliations:** † Department of Physics, 58207 Hong Kong University of Science and Technology, 999077 Hong Kong, China; ‡ William Mong Institute of Nano Science and Technology, Hong Kong University of Science and Technology, 999077 Hong Kong, China

## Abstract

The mechanical relaxation behavior of freestanding poly­(methyl methacrylate) (PMMA) films and freestanding PMMA supported by a polydimethylsiloxane micrometer film was investigated by using dynamic mechanical analysis (DMA). Results reveal two tiers of enhanced molecular mobility near the surface: a significant enhancement in the nanoscale outer region and a lesser enhancement in a thicker region (thickness, *h*_t_ ∼ 140 nm) underneath, consistent with observations made in polystyrene (PS) films, where *h*_t_ ∼ 1000 nm, however. Coarse-grained molecular dynamics simulations of PMMA and PS suggest that fast-moving molecules in the nanoscale surface region activate adjacent molecules, facilitating collective motion in both polymers. For PMMA, this enhancement terminates at a distance consistent with the experimental observation. In contrast, for PS, this dynamic enhancement persists up to the simulated thickness of 250 nm, showing no sign of termination. These findings support the role of near-surface collective motions in driving long-range mobility enhancement, with greater enhancement observed in PS compared to PMMA, accounting for the different *h*_t_ values.

## Graphical abstract

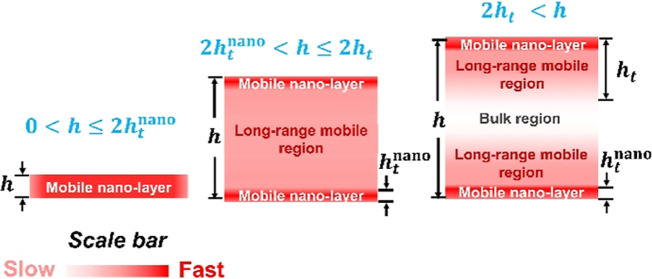

## 1 Introduction

Polymer thin films are broadly utilized in scientific and industrial applications, including flexible electronics,^1–3^ food packaging,^4^ and organic optoelectronic devices.^5–7^ The glass transition behavior of these films is a critical factor that influences their service life and informs technical guidance for practical applications.^8,9^ Studies show that the near-surface region of polymer glass films typically exhibits higher free volume^10–14^ and lower molecular chain packing density.^15,16^ These characteristics facilitate the relaxation of surface chain segments, resulting in significantly higher mobility of the surface molecules^17–21^ compared to bulk molecules. This phenomenon, known as the free surface effect, is often equated with the existence of a “liquid-like” or mobile surface layer.^17,19,22–24^

The layer model is the most commonly used framework for describing the dynamic distribution in thin polymer films.^19,24^ It depicts the films as comprising a mobile surface layer and an underlying layer with lower, bulk-like mobility. As the thickness (*h*) of the film decreases, the mobile surface layer progressively dominates the relaxation behavior of the film. Enhanced molecular mobility has been shown to significantly influence the mechanical relaxation,^17,25^ dielectric relaxation,^26–28^ and glass transition^10,15,24,29,30^ characteristics of thin polymer films. Therefore, investigating the length scales associated with the free surface effect is crucial for understanding relaxation behavior and optimizing performance.

Numerous studies have demonstrated the role of the free surface effect in promoting molecular dynamics (MD) within the nanometer-scale near-surface region. For example, Yang et al.^24^ measured the effective viscosity of unentangled short-chain PS films of varying thicknesses by monitoring surface morphology changes using atomic force microscopy (AFM) during annealing. Their findings confirmed the existence of a mobile surface layer approximately 2.3 nm thick in PS films. Chai et al.^31^ studied the evolution of a step profile created in PS films at temperatures below *T*_g_, which revealed a similar mobile surface layer. Teichroeb et al.^32^ used AFM to study the embedding of gold nanoparticles on PS surfaces and found that the top 3–4 nm of the film remains in a rubbery state at temperatures below *T*_g_. However, several previous studies employing macroscopic methods^17,25,33–36^ have indicated that deviations from bulk dynamics begin at microscale film thickness rather than nanoscale thickness. A recent experiment^17,25^ provided crucial insights by demonstrating that the surface dynamics of polystyrene (PS) are heterogeneous, comprising a nanoscale mobile surface layer and a microscale sublayer beneath it.

In this study, we use dynamic mechanical analysis (DMA) to study the relaxation behaviors of freestanding PMMA and freestanding PMMA–polydimethylsiloxane (PDMS) bilayer films with thicknesses ranging from 4 nm to 48 μm. Consistent with previous investigations of PS with and without a PDMS support,^17,25^ the presence or absence of a soft PDMS substrate is also found to have no impact on the modulus (*E*) and relaxation time (τ) of the PMMA polymer. Additionally, our results show that PMMA has a mobile surface layer with a bilayer structure similar to our earlier observation in PS.^17^ However, its long-range mobile sublayer is thinner, measuring ∼140 nm.

To explore the mechanism underlying this enhancement in long-range dynamics, we employ a coarse-grain (CG) molecular model to simulate the surface mobile bilayer in both PMMA and PS. The simulation results reveal that movements of the near-surface molecules drive adjacent molecules beneath to move coherently toward the same direction, similar to the effects observed under shear excitation. This enhanced mobility from the surface persists up to approximately 140 to 155 nm for PMMA and more than ∼250 nm for PS, in agreement with experimental findings.

## 2 Materials and Methods Section

### 2.1 Materials

Poly­(methyl methacrylate) (PMMA), with a weight-average molecular weight (*M*_w_) of 1637 kg/mol and a polydispersity index (PDI) of 1.05, was purchased from Scientific Polymer Products (Ontario, NY). Before use, PMMA was dissolved in toluene and filtered through a 0.22 μm PTFE membrane without further purification. PDMS (SYLGARD 184), comprising a base precursor and a curing agent, was purchased from Dow Corning. Silicon wafers (100) with a native oxide (SiO_*x*_) layer were purchased from University Wafer Inc., diced into approximately 1.5 × 1.5 cm^2^ pieces, submerged in a piranha solution at 130 °C for 20 min, and thoroughly rinsed with deionized water before use.^37^ Mica sheets (1 in. × 1 in.) were purchased from TED PELLA, Inc., and used as substrates during the fabrication of freestanding films.

### 2.2 Preparation of Freestanding (Uncapped) and One-Side SiO_*x*_-Capped PMMA Films

Thin PMMA films (*h* < 1 μm) were prepared by spin-coating toluene solutions with polymer concentrations between 0.5% and 5% by mass onto freshly cleaved mica at 3000 rpm for 30 s. All films were annealed under a vacuum at 403 K (*T*_g_ + 15 K) for 12 h to remove residual solvent and processing histories. To minimize thermal stress during cooling, the films were cooled slowly under vacuum at a rate of approximately 0.5 K/min to room temperature. Freestanding PMMA films were created using a water transfer technique,^17,25^ where the PMMA films were transferred from mica substrates onto polyethylene terephthalate (PET) supporting frames with a 1 cm × 1 cm square opening.^17,25^ These freestanding films were dried naturally under ambient conditions and then cut into 4 mm wide strips with a laser cutter. Similarly prepared PMMA films were also fabricated on cleaned Si substrates to determine their thickness using a single-wavelength (632.8 nm) ellipsometer (LSE Stokes Ellipsometer, Gaertner Scientific Corp.), following the method outlined in ref 38.

Thicker PMMA films with *h* > 1 μm were prepared by solution casting in a customized Teflon mold (3 cm × 4 cm area). The filtered PMMA/toluene solution was placed in the mold and allowed to evaporate in a controlled ambient environment for 24 h. The PMMA films in the molds were then annealed at 403 K for 24 h and slowly cooled under a vacuum to ambient temperature. After demolding, the PMMA films were cut into 2 mm strips by using a razor blade. The film thickness was controlled by the mass of dissolved PMMA. Notably, the thickness-dependent relaxation measurements of these films shown below (Figure 6b,c) indicate no visible discontinuity or qualitative changes at *h* = 1 μm. This clearly demonstrates that the relaxation behaviors of our films are independent of the film preparation method.

One-side silica-capped PMMA films (*h* > 500 nm) were prepared by depositing a ∼150 to 120 nm thick silica (SiO_*x*_) layer onto mica substrates coated with a poly­(acrylic acid) sacrificial layer before spin-coating the PMMA films. The thickness of the SiO_*x*_ layer was determined by measuring the profile of a scratched region using a scanning probe microscope (SPA-300HV, Seiko Instrument Inc.). PMMA films spin-coated onto the SiO_*x*_ layer were annealed at 403 K for 12 h, promoting adhesion between the SiO_*x*_ layer and the PMMA film and removing the processing history. Following a slow cooling under vacuum to ambient temperature (ca. 0.5 K/min), the PMMA–SiO_*x*_ bilayers were transferred onto PET supporting frames using the water transfer technique and cut into 4 mm strips using a laser cutter. The successful transfer of the PMMA–SiO_*x*_ bilayer was confirmed by atomic force microscopy (AFM), which showed that the mica substrate remained unchanged before SiO_*x*_ deposition and after water transfer.

### 2.3 Preparation of PDMS Supporting Layer and PMMA–PDMS Bilayer

To prepare the PDMS supporting layer, the Sylgard 184 base precursor and curing agent were combined in a 10:1 mass ratio and thoroughly mixed by using a vortex mixer. After degassing the mixture for 30 min, PDMS films were fabricated by spin-coating onto Petri dishes using a two-step process: an initial spread at 1000 rpm for 10 s, followed by spinning at 3000 rpm for 60 s. The PDMS films were cured at 343 K for 2 h and subsequently cut into 4 mm wide strips using a razor blade. Films prepared using this protocol had a thickness of approximately 20 μm and elastic modulus (*E*_PDMS_) of ∼0.9 to 1 MPa, consistent with previously reported values.^25^ To assemble the PMMA–PDMS bilayer, a PDMS strip was slightly stretched and mounted onto a rigid supporting frame by draping it across a 1 × 1.5 cm^2^ rectangular opening. Subsequently, an annealed PMMA film (thickness, *h* < 500 nm) was floated onto the surface of deionized water and then carefully transferred onto the premounted PDMS film. After trimming any excess PMMA, the assembled PMMA–PDMS bilayer was dried under vacuum at room temperature for 12 h. No postannealing was performed afterward to preserve the integrity of the bilayer structure.

### 2.4 Study of the Effect of Strain Rate (γ̇) and Film Thickness (*h*) on the Elastic Modulus of PMMA Films (*E*)

The strain rate (γ̇)-dependent elastic modulus of PMMA thin films (*h* < 200 nm), E(γ̇) , was determined by measuring the stress–strain curves of PMMA–PDMS bilayers at different γ̇ using a DMA (Q800, TA Instruments). The elastic moduli of the PDMS supporting layer (*E*_PDMS_) and the PMMA–PDMS bilayer (*E*_bilayer_) were separately determined from the slope of their stress–strain curves. Using a PDMS layer thickness of *h*_PDMS_ = 20 μm and its elastic modulus value, *E* was calculated using the following equation:^17,25^

1 $$E=\frac{1}{h}\left[\left(h+h_{PDMS}\right)\cdot E_{bilayer}-h_{PDMS}\cdot E_{PDMS}\right]$$

Our previous study showed that *E*_PDMS_ exhibits no dependence on strain rate with similar measurements.^17^ Therefore, any strain rate dependence observed in the PMMA–PDMS bilayer must originate from PMMA itself. As we will discuss further, both the elastic modulus and relaxation time of the PMMA layer remain independent of whether it is supported by PDMS or is freestanding. This independence is attributed to the lack of annealing in the PMMA–PDMS bilayer, which is necessary for the establishment of dynamic coupling between the two layers.^39^ From our experience, this method is applicable only to films thinner than ∼200 nm. To investigate the relaxation behavior of thicker films, we utilized the method detailed in the next section.

### 2.5 Stress Relaxation Measurements of Freestanding PMMA Films

Stress relaxation measurements of freestanding PMMA films were performed using the stress relaxation mode of the DMA. After mounting, the film was heated to the target temperature at a rate of 15 K/min and held isothermally for 10 min if the target temperature was more than 5 K below *T*_g_, or 2 min if it was within 5 K below *T*_g_, to ensure temperature equilibrium. Subsequently, a small tensile strain (γ ∼ 0.5%) was applied, and the elastic relaxation modulus, *E*(*t*), was recorded as a function of time. Stress relaxation of the film was allowed to occur for ca. 1500–2000 min^17^ at varying temperatures (*T*) between room temperature and 387 K (cf. *T*_g_ of PMMA is ∼388 K). We found that this measurement protocol was effective across a broad range of film thicknesses, from ∼100 nm to 48 μm, with no special procedures needed for studying the nanometer-thick films. While the method remains effective for films thicker than 4 μm, data quality begins to deteriorate for films thinner than ∼100 nm.

### 2.6 Coarse-Grain Molecular Dynamics Simulations of Polymer Films

We use a CG molecular model, as employed by Hsu et al.^40–42^ to investigate the near-surface dynamics in polymer glass films. Our focus of this work is on examining molecular displacements that maintain coherence persisting over long distances from the surface, as well as comparing the dynamics of PS and PMMA polymers. In our model, each polymer is represented as consisting of one backbone (A) and one side-group (B) pseudo atom within each monomer, with molecular representations shown in Figure 1a. For PS, the model considers the phenyl ring as the side-group, while for PMMA, the side-group is represented by the methyl group. The latter choice indicates a division between the main chain and the side chain at the C–O bond, which is supported by vibrational density of states data, indicating that the C–O bond is the most flexible bond in PMMA.^42^ Figure 1b illustrates a simulated polymer chain (upper panel) and the simulation setup used to investigate MD near the surface of the films (lower panel). The blue and red dots represent the backbone and side-group atoms, respectively. The film thickness (*L*_*z*_) is 23 nm, with lateral dimensions of *L*_*x*_ = *L*_*y*_ = 55 nm. The model includes 2209 chains for PMMA and 1800 chains for PS, with each polymer chain containing 200 monomers. Figure 1c presents another simulation setup used to examine molecular displacements with long-range coherence along the *z* direction. This model has a thickness of 250 nm and a smaller cross-section of 9 × 9 nm^2^ due to computational constraints.

**Figure 1:**
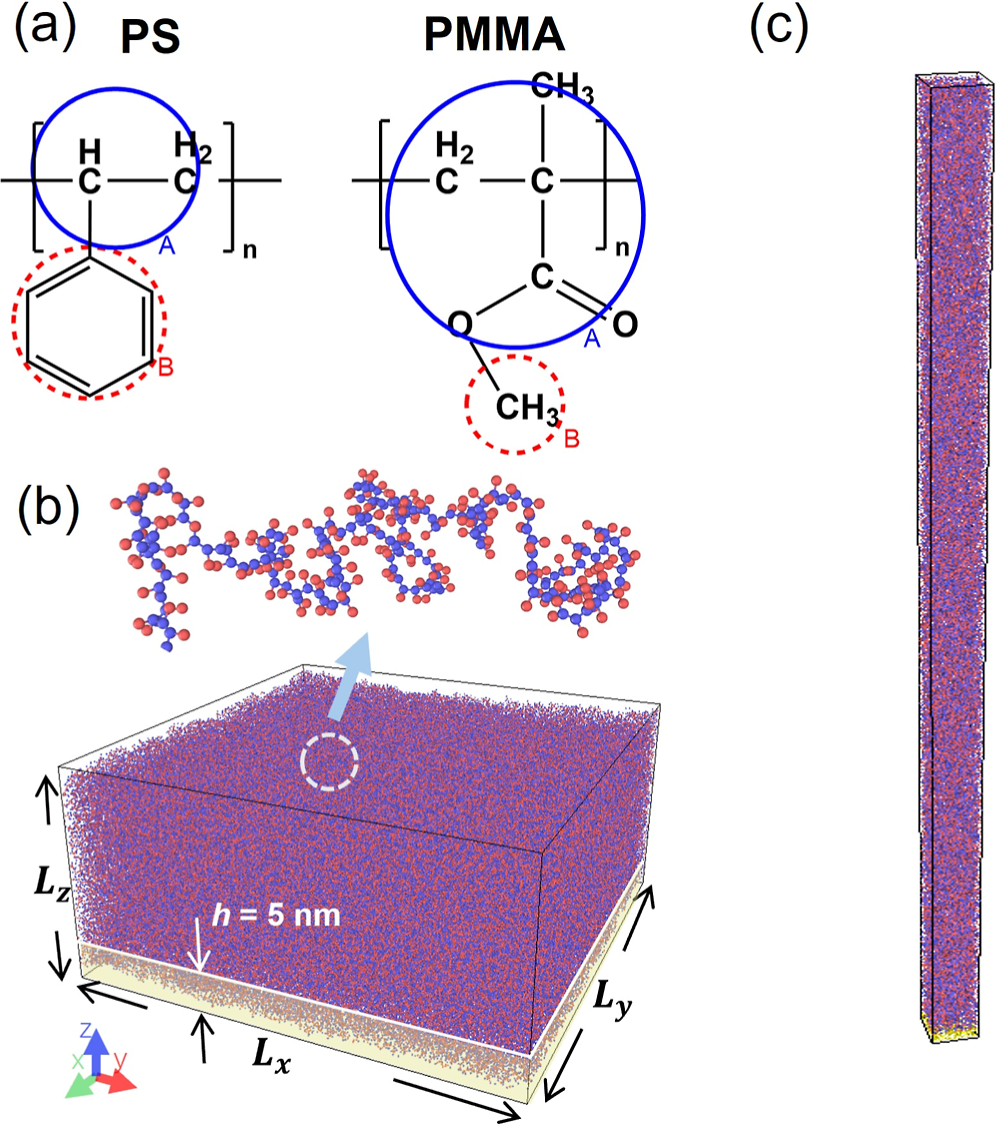
Coarse-grain (CG) molecular dynamics (MD) simulations of the mobile surface region in PS and PMMA films. (a) Chemical structures of PS and PMMA along with their CG models, where the backbone and side-group pseudo atoms are represented by blue and red circles, respectively. (b) Snapshot of the simulation setup investigating the MD of the polymers, including the free surface. (c) The slender CG model setup used to investigate long-range coherence along the *z* direction.

Both simulation setups employ periodic boundary conditions in the *x* and *y* directions. To prepare the system, we followed a protocol developed by Keten’s group.^40–42^ Specifically, the initial structures, generated using a random walk algorithm, are annealed using the open-source package Large-scale Atomic/Molecular Massively Parallel Simulator (LAMMPS),^43^ employing a specific force field published by this group^40^ to achieve the target density. The potential energy of the system is then minimized by using the conjugate gradient algorithm. During this minimization, we first perform a soft potential push-off step before applying the full force field. Then the model undergoes three annealing cycles, where the temperature is increased from 300 to 750 K at 4.5 K/ps, followed by a cooling phase back to 300 K at the same rate. A Nose–Hoover barostat is applied at 10,000 atm for 2.5 ns to enhance density convergence. After the high pressure was alleviated and the dynamics were simulated at 300 K and 1 atm under the NPT ensemble for 2 ns, no residual stresses are observed. This cycle ensures that the local structure and thermodynamic properties are independent of the initial configuration. In this protocol, a time step of Δ*t* = 4 fs, a temperature damping parameter of *T*_damp_ = 400 fs, and a pressure damping parameter of *P*_damp_ = 1000 fs were used. These protocol parameters have been confirmed to effectively map realistic atomistic models for various molecular and macroscopic properties, including bond distributions, characteristic ratio, mass density, *T*_g_ and its molecular weight dependence, and so on.

The model in Figure 1b is used to simulate the mobility gradient from the free surface to the inner bulk region.^44,45^ The chosen model thickness (*L*_*z*_ = 23 nm) is constrained by resource limitations. To account for the macroscopic thickness in the *z* direction in the experiment, an additional frictional force is applied to the atoms within the bottom yellow region (Figure 1b), which has a thickness of 5 nm. This frictional force (*f*) is proportional to the instantaneous velocity (*v*): *f* = −λ*v*, where λ is set to 0.5 for both PMMA and PS. This choice of λ is supported by the observation that variations of ± 0.2 (a 40% change) do not noticeably affect the root-mean-square velocity (*v*_rms_) at the surface. Further details can be found in the Supporting Information.

For the model in Figure 1c, a substrate consisting of heavy atoms (with a molar mass of 10^7^ g/mol; in comparison, the molar mass of the A and B atoms of the PMMA and PS polymers studied ranges from ∼15 to ∼85 g/mol), arranged in a face-centered cubic, with a lattice constant of $4\sqrt{2}\;Å$ , is implemented in the bottom yellow region, with a thickness of 20 nm. We chose this model over one with two free surfaces to avoid complexity that may arise from interactions between two near-surface mobility gradients. We believe such interactions are likely due to the long-range nature of the mobile surface layer observed in this and previous experiments.^17^

The Lennard–Jones potential is employed for the interactions between CG atoms and the substrate:

$$V_{LJ}\left(r\right)=4\varepsilon \left[{\left(\frac{\sigma }{r}\right)}^{12}-{\left(\frac{\sigma }{r}\right)}^{6}\right]$$

where σ = 4 Å and ε = 1 kcal/mol, close to those in the polymers. The less restrictive bottom boundary condition in the model shown in Figure 1b is implemented to prevent the substrate’s influence on the motion of the surface atoms due to the smaller thickness of 23 nm, as confirmed by the data shown in Figure S3. Unless specified otherwise, vertical position *z* refers to the location of atoms relative to the upper edge of the bottom yellow region.

MD simulations were performed using LAMMPS.^43^ The Nosé–Hoover thermostat was employed to control the temperature, with a time step of 4 fs. The initial model was equilibrated at 300 K following the procedure described by Hsu et al.^40–42^ Then, the model was heated to 1.35 *T*_g_ and equilibrated at this temperature for 10 ns for the model shown in Figure 1b and 200 ns for the model shown in Figure 1c. During this process, physical quantities, such as position and velocity, were recorded every 0.4 ns for further analysis.

## 3 Results and Discussion

### 3.1 Mobile Surface Nanolayer: Study of Thickness Dependence of the Mean-Film Relaxation Time, τ­(*h*)

Figure 2a shows the dependence of the normalized elastic modulus, *E*(γ̇)/*E*_max_, of the PMMA layer on the strain rate (γ̇) and film thickness (*h*) at room temperature. Here, *E*_max_ represents the maximum modulus value, as provided in Table S1. The fact that these values are comparable to those exhibited by the PMMA freestanding films at similar temperatures (Figure 3) demonstrates that the PDMS supporting layer does not influence the elastic modulus of the PMMA layer. As γ̇ increases, *E*(γ̇)/*E*_max_ increases and plateaus at 1 when γ̇ exceeds a characteristic strain rate, which increases as *h* decreases. As demonstrated in our previous works,^17^ this characteristic γ̇ is indicative of the mean relaxation time (τ) of thin films. When γ̇ < τ^–1^, *E* decreases due to the segmental relaxation of polymer chains under strain. Conversely, when γ̇ > τ^–1^, the chain motions cannot keep pace with deformation, causing *E* to approach its maximum value of *E*_max_. The γ̇-dependence of *E* can be modeled using the following stretched exponential function^25^ (eq 2):

2 $$E\left(\mathrm{\gamma ̇}\right)/E_{\max}=1-\exp\left[-{\left(\mathrm{\gamma ̇}\tau \right)}^{\beta }\right]$$

where β is the stretching exponent, reflecting the dynamic heterogeneity of the system.^46^ By fitting the experimental data in Figure 2a to eq 2, the τ and β values were determined.

**Figure 2:**
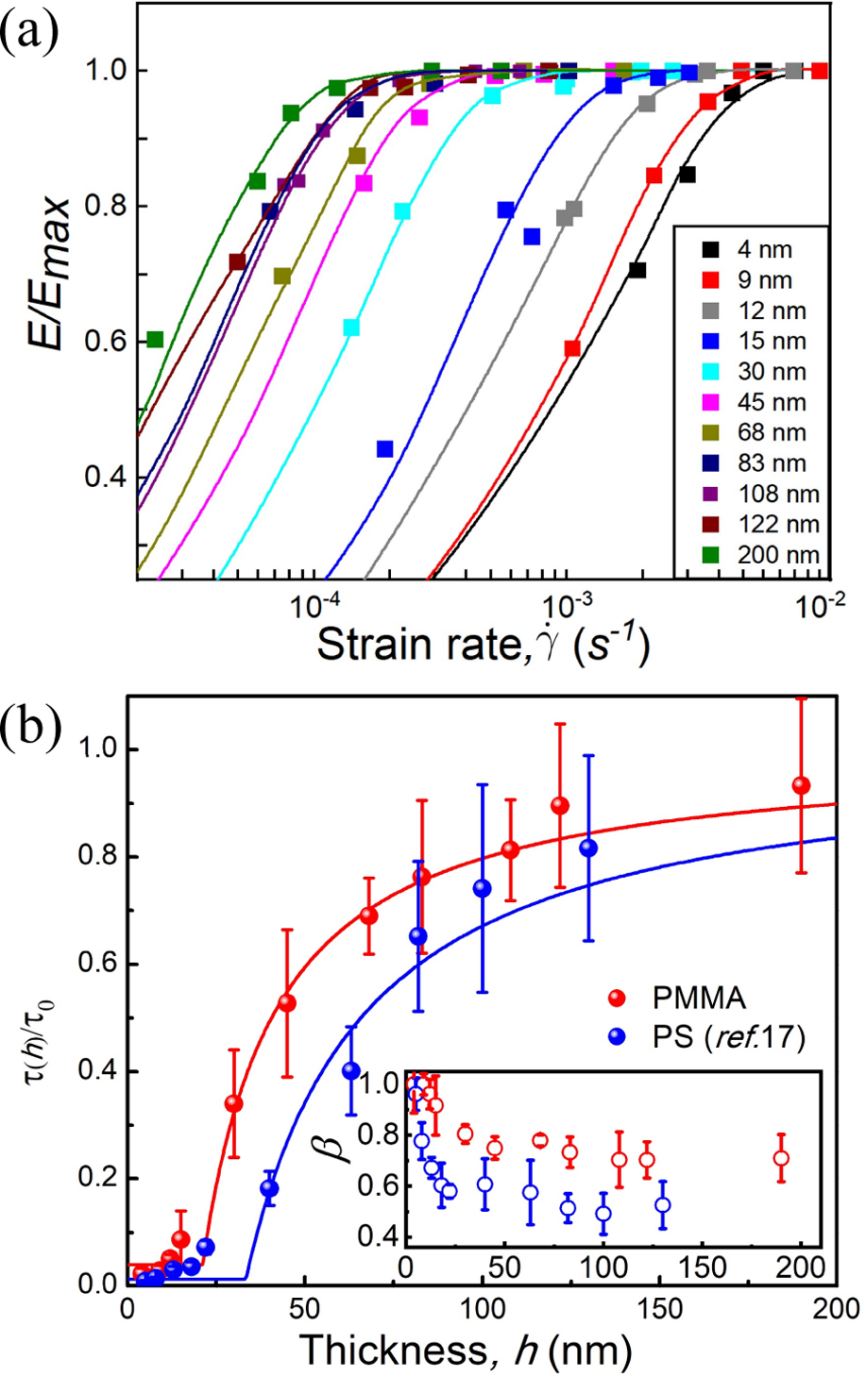
(a) Room-temperature normalized elastic modulus (*E*/*E*_max_) versus strain rate (γ̇) of PMMA films supported on PDMS with various PMMA thicknesses (*h*). The solid lines represent the best fit to eq 2. (b) Semilogarithmic plot of the normalized relaxation time (τ/τ_0_) derived from the data of (a) as a function of *h*. Previous results for PS films are also shown (blue symbols), reproduced from ref 17. Available under a CC-BY license 4.0. Copyright 2022, Yuan et al. The solid lines represent the best fits of τ/τ_0_ to eq S1, assuming a Heaviside step function profile for τ. The inset shows a plot of β versus *h*.

Figure 2b presents the normalized mean-film relaxation time (τ/τ_0_) for PMMA films as a function of *h*, alongside previous data for PS films for comparison.^17^ Here, τ_0_ represents the extrapolated saturation relaxation time at large *h*, determined by fitting the data of τ­(*h*) versus *h* to eq S1 before normalization, with further details provided below. As *h* increases from 4 nm, τ/τ_0_ for both PS and PMMA changes relatively gradually, attributed to significantly reduced surface relaxation times (τ_surf_) for thicknesses below a nanoscale critical threshold, beyond which τ/τ_0_ increases and approaches a plateau region as *h* exceeds 100 nm. The inset of Figure 2b shows β as a function of *h* for both PS and PMMA films. Notably, β decreases from 1 as *h* increases, signifying a broader distribution of relaxation times and an increased dynamic heterogeneity in thicker films.^46,47^ These findings support the presence of a mobile nanoscale layer at the surface, beneath which the dynamics slow down and become glassy, with τ approaching τ_0_ as distances exceed ∼100 nm. To estimate the threshold thickness and the values of τ_0_ and τ_surf_, we employ a layer model for freestanding films (eq S2), where a central bulk-like layer is sandwiched between two mobile surface layers (Figure S1a). For simplicity, we assume homogeneous dynamics within each layer and use eq S1 for the relaxation time profile τ­(*z*). We then calculate τ­(*h*) by taking a linear arithmetic average of the contributions from each layer, as specified in eq S2. The solid lines in Figure 2b represent fits of this model to the experimental τ data, followed by normalization by τ_0_. The fit values of τ_surf_ for PMMA and PS films are ≈1000 and 3000 s, respectively, which are substantially shorter than their bulk relaxation times (see Figure 4 and refs 17, 48, and 49 ), indicating a surface-induced mobility enhancement within the nanoscale surface region. Notably, the τ_0_ values, being ∼3 × 10^4^ s for PMMA and ∼2.3 × 10^5^ s for PS, are also much shorter than the respective bulk relaxation times.^17,48,49^ This suggests that the dynamics in the region adjacent to the mobile surface nanolayer, even at distances greater than 100 nm beneath, are still considerably faster than the bulk dynamics. We further note that the threshold thickness, corresponding to twice the thickness of the mobile surface nanolayer, 2*h*_t_^nano^, is slightly larger for PS (∼32 nm) than that for PMMA (∼22 nm).

**Figure 3:**
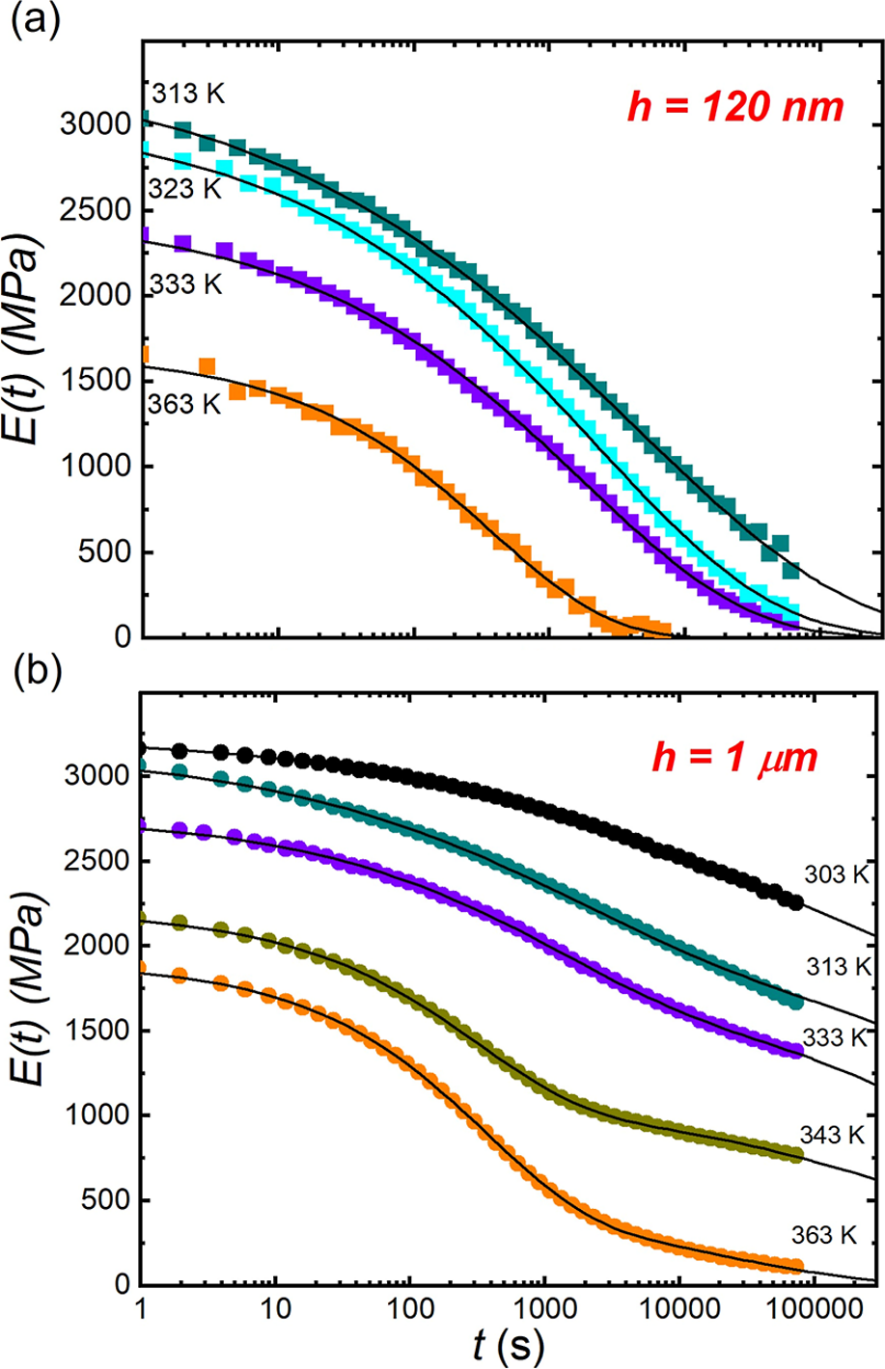
Relaxation modulus curves of PMMA films at different temperatures: (a) 120 nm and (b) 1 μm. The solid lines represent the best fits to the double KWW function (eq 3). In (a), all curves exhibit single exponential decay with *E*_2_ = 0.

**Figure 4:**
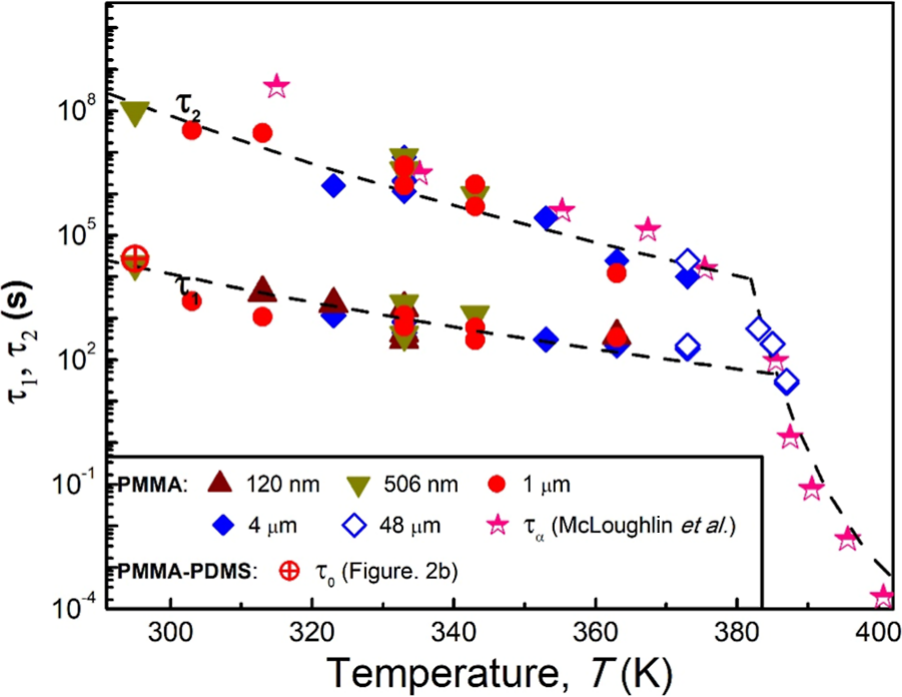
Plots of the relaxation times, τ_1_ (fast relaxation mode) and τ_2_ (slow relaxation mode), of freestanding PMMA films at different temperatures *T*. The saturation relaxation time (τ_0_) of PMMA supported on PDMS at *T* = 295 K, deduced from Figure 2b, is also included (red ringed cross). For comparison, the bulk structural relaxation time, τ_α_, measured by McLoughlin and Tobolsky is shown (half-filled stars). Adapted with permission from ref 49. Copyright 1952, Elsevier Science Ltd. The black dash lines represent Arrhenius fits to τ_1_ and τ_2_ at temperatures below 380 K, and the VFT fit for τ_2_ at temperatures above 380 K.

### 3.2 Propagation of Surface-Enhanced Mobility beyond the Surface Nanolayer

To investigate the length scale at which τ returns to the bulk relaxation time, we performed stress relaxation experiments on films with *h* greater than 100 nm. Figure 3a,b displays the time-dependent relaxation modulus, *E*(*t*), for freestanding PMMA films with *h* = 120 nm and 1 μm, respectively, measured at various temperatures *T*. The results show that the initial relaxation modulus (*E*_0_) decreases as *T* increases from 313 to 363 K, indicating softening of the polymer as expected. Regarding the shape of the *E*(*t*) curves, those for the 120 nm films display a single exponential decay, while the 1 μm films show a step-like feature, indicating that the 120 nm films relaxed by one relaxation mode and the 1 μm films by two modes. We fitted all *E*(*t*) curves using the double Kohlrausch–Williams–Watts (KWW) function:

3 $$E\left(t\right)=E_{1}\exp\left[-{\left(\frac{t}{\tau_{1}}\right)}^{\beta_{1}}\right]+E_{2}\exp\left[-{\left(\frac{t}{\tau_{2}}\right)}^{\beta_{2}}\right]$$

Here, the subscripts (*i*) of 1 or 2 denote the fast or slow relaxation mode, respectively. *E*_*i*_, τ_*i*_, and β_*i*_ represent the initial relaxation modulus, relaxation time, and stretching exponent for the mode *i*. When only one relaxation mode is responsible for the relaxation process, such as for the 120 nm films (Figure 3a), the contribution of the *i* = 2 mode is zero (with *E*_2_ = 0), and eq 3 is simplified to the single KWW function. The solid lines in Figure 3 denote the best fit to eq 3.

Figure 4 displays the fast (τ_1_) and slow (τ_2_) relaxation times for PMMA films, with *h* = 120 nm, 560 nm, 1 μm, 4 μm, and 48 μm plotted as a function of temperature *T*. Except for the 120 nm films, all of the thicker films possess both fast and slow relaxation times. Notably, τ_1_ for these thicker films agrees with the relaxation time of the 120 nm films. The half-filled stars represent the bulk α-relaxation time (τ_α_) measured by McLoughlin and Tobolsky.^49^ As shown, these data align with τ_2_ in an overlapping temperature range. For temperatures above 380 K, both data sets exhibit Vogel–Fucher–Tammann (VFT) temperature dependence, characteristic of α-relaxation. This observation of τ_2_’s VFT behavior strongly supports its association with α-relaxation. Below 380 K, however, the behavior transitions to Arrhenius behavior. The reason for this change remains unclear. Casalini and Roland previously suggested that it may be due to the freezing of the polymer’s structure below *T*_g_.^50^ Interestingly, this suggestion aligns with recent simulation findings^15^ indicating that nonequilibrated polymer systems display a similar transition in their α-relaxation time–temperature dependence. After recoginzing τ_2_'s association with α-relaxation, we now focus on exploring the origin of the fast relaxation mode (τ_1_).

Recently, much attention has been directed toward the Slow Arrhenius Process (SAP) reported by Song et al.^51^ These authors measured the relaxation times associated with this process (τ_SAP_) using dielectric spectroscopy, but only at temperatures above *T*_g_, where τ_SAP_ was found to be longer than the α-relaxation. Below *T*_g_, τ_SAP_ could not be measured directly but is believed to be involved in various processes, including aging and adsorption, that the polymer undergoes to equilibrate. The authors argue that the defining characteristic of SAP below the *T*_g_ is the activation energy (*E*_a,SAP_), rather than the time scale of the equilibration processes (τ_eq_). To investigate whether our fast relaxation mode (τ_1_) is related to this SAP, we created separate plots of τ_1_ versus *T* for PMMA and PS in Figure 5a,b, respectively, represented by black lines. We also included the τ_SAP_ data from Song et al.,^51^ denoted by brown diamonds and accompanied by brown dashed Arrhenius fit lines. We analyzed the activation energies *E*_a,1_ and *E*_a,SAP_ for τ_1_ and τ_SAP_ and presented their values in Table 1. Our comparison reveals that *E*_a,1_ and *E*_a,SAP_ do not align for both PS and PMMA.

**Figure 5:**
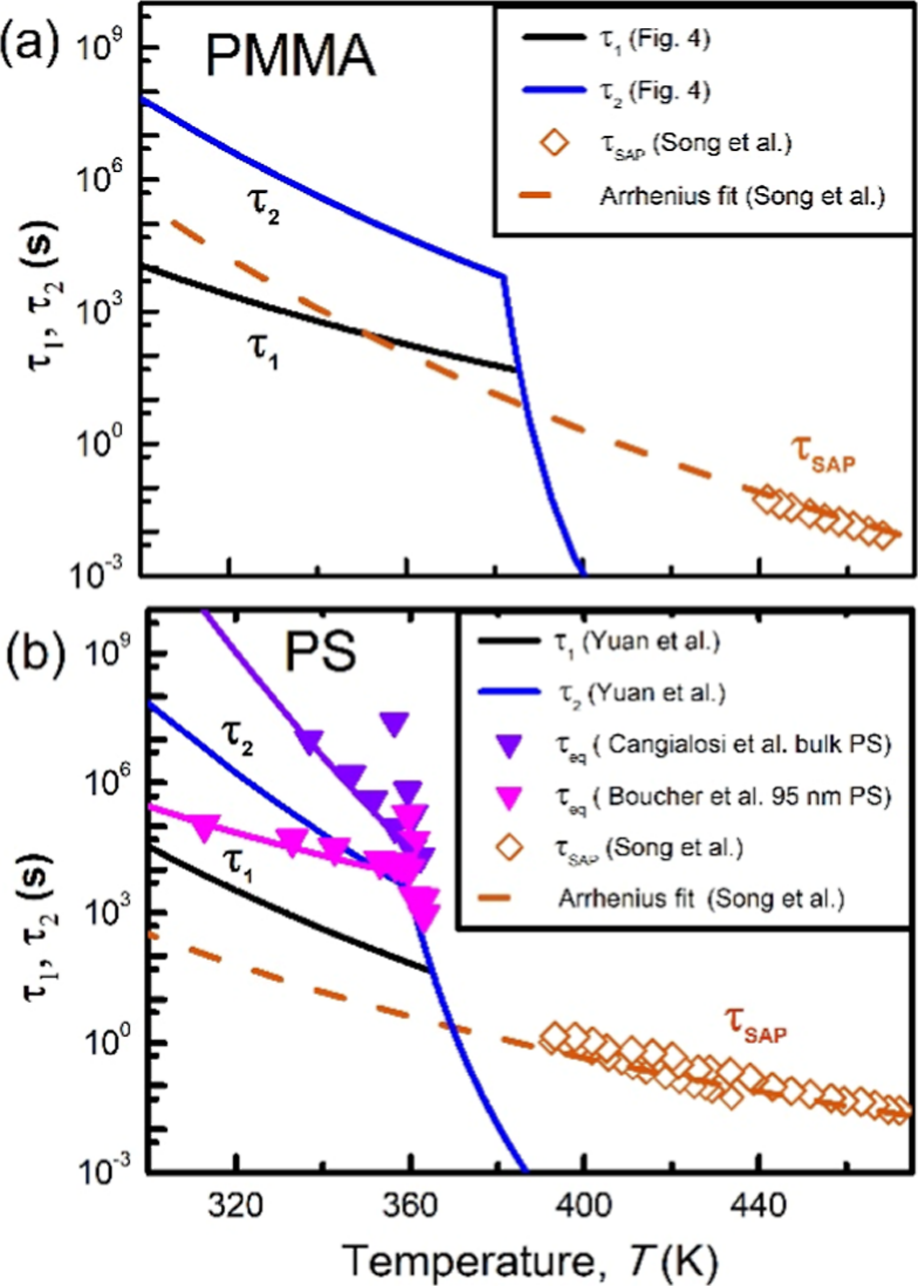
Comparison between the temperature dependences of τ_1_ (depicted by thick black lines) and τ_2_ (depicted by thick blue lines) for PMMA from Figure 4 (a) and for PS from our group in ref 17 (b) with those of the slow Arrhenius relaxation time (τ_SAP_) data (brown diamonds and dashed line), adapted from ref 51. Available under a CC BY-NC 4.0 License. Copyright 2022, Song et al. In (b), data from enthalpy recovery are also included. Adapted with permission from ref 52 (purple inverted triangles), Copyright 2013, American Physical Society, and ref 53 (magenta inverted triangles), Copyright 2017, American Institute of Physics.

**Table 1:** Activation Energies from Arrhenius Fits to τ_1_, τ_SAP_, and Relaxation Times from Enthalpy Recovery (refs 52,53 )

polymer	PS	PMMA
*E*_a,1_ (kJ/mol)	91 ± 6^a^	62 ± 8
*E*_a,SAP_ (kJ/mol) (ref 51)	69 ± 6	119 ± 3
*E*_a,bulk,fast_ (kJ/mol) (ref 52)	252 ± 26	
*E*_a,95 nm,fast_ (kJ/mol) (ref 53)	55 ± 9	

a Yuan et al.^17^

By investigating enthalpy recovery in bulk PS, Cangialosi et al.^52^ also observed two relaxation modes, which align with our findings. When comparing their relaxation time versus temperature data with our τ_1_ and τ_2_ (black line and blue line, respectively, Figure 5b), we note that their slow relaxation time aligns with an extension of the VFT relation of our τ_2_ at high temperature. At lower temperatures (<∼360 K), their fast relaxation time displays an Arrhenius temperature dependence, with a reduced activation energy (*E*_a,bulk,fast_). This value, as shown in the third row of Table 1, is greater than both *E*_a,1_ and *E*_a,SAP_. In a subsequent study, Boucher et al.^53^ found that for films with a thickness of 95 nm, the activation energy of the fast mode becomes smaller at low temperatures. We observe that this activation energy value (*E*_a,95 nm,fast_) deviates from *E*_a,1_ but aligns with *E*_a,SAP_ (see Table 1). Based on the above comparisons, there is no clear evidence to suggest a connection between our fast relaxation mode and SAP; however, a link may exist between the fast mode observed by Boucher et al. and SAP.

Insights into the origin of τ_1_ emerge when we compare the τ_0_ value from Figure 2b (denoted by the red ringed cross in Figure 4) with the other data in Figure 4. This comparison indicates a strong alignment between τ_0_ and τ_1_, suggesting that the fast relaxation mode correlates with the near-surface relaxation time (τ_0_). This relationship is further clarified when we examine the thickness-dependent relaxation behavior of PMMA films at a fixed temperature of *T* = 333 K. Figure 6a shows that as *h* decreases from 4 μm to 120 nm, the *E*(*t*) stress relaxation curve transitions from double KWW to single KWW behavior. Concomitantly, the contribution of the fast mode to the overall relaxation dynamics, *E*_1_(*t*)/*E*(0) (dashed lines) increases. Using the simplest layer model for freestanding films, where the relaxation time of the middle layer has the bulk value^23,24^ and fast relaxations occur in the outer mobile surface layer with thickness *h*_t_ and are uniform, we can express the fraction of the fast component, *f*_1_, by

4 $$f_{1}\equiv \frac{E_{1}}{E_{1}+E_{2}}=\frac{2h_{t}}{h}$$

**Figure 6:**
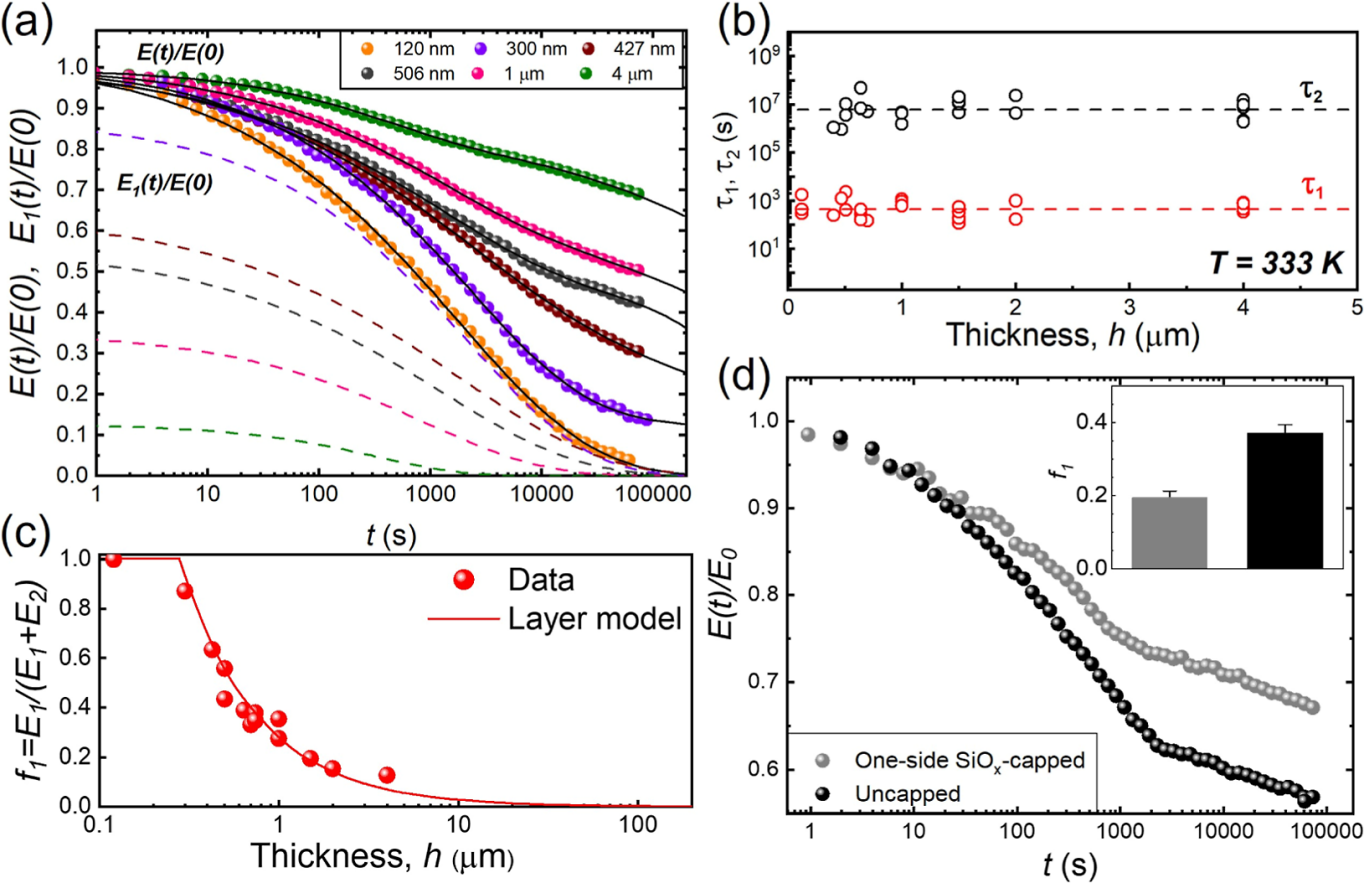
(a) Normalized relaxation moduli, *E*(*t*)/*E*(*0*), of freestanding PMMA films with different *h* at *T* = 333 K as a function of time *t* (symbols). Solid lines are fits to the double KWW function (eq 2), while dash lines represent the normalized fast relaxation components, *E*_1_(*t*)/*E*(*0*), deduced from these fits. (b) Plots of τ_1_ and τ_2_ at 333 K versus *h*. (c) Plot of the fast component fraction, *f*_1_ = *E*_1_/(*E*_1_ + *E*_2_) versus *h*. (d) Plots of *E*(*t*)/*E*(*0*) of uncapped and one-side SiO_*x*_-capped 740 nm PMMA films as a function of time. Inset: the fast relaxation strength *f*_1_ for both uncapped (black bar) and one-side SiO_*x*_-capped (gray bar) 740 nm PMMA films. The *f*_1_ measurements and error bars are determined by the average value and standard deviation from multiple measurements.

Figure 6c displays the variation of *f*_1_ as a function of film thickness *h* for PMMA at *T* = 333 K. The solid line, representing the best fit to eq 4, describes the data well. It reveals that the 2*h*_t_ value for PMMA is ∼280 nm, implying an *h*_t_ value of 140 nm. This obtained *h*_t_ value is unexpectedly large and is not accountable by any existing theories.

To confirm that this long-range fast relaxation mode originates from the free surface, we examined whether the fast mode disappears when the free surface effect is suppressed. In our previous study on PS,^17^ a gold layer ∼150 nm thick was deposited onto the free surfaces of PS films, suppressing the free surface effect through strong gold-PS interactions.^17,54,55^ Here, we suppress the free surface effect in PMMA through PMMA–SiO_*x*_ interactions. Keddie et al.^23^ found that the *T*_g_ of PMMA films supported by SiO_*x*_-coated Si increases as *h* decreases, indicating that the PMMA–SiO_*x*_ interactions are strong. We recently demonstrated that these interactions can slow down the segmental dynamics at the free surface in thin PMMA supported by SiO_*x*_ with *h* < 27 nm.^55^ Figure 6d shows the *E*(*t*) relaxation curves for uncapped and one-side SiO_*x*_-capped freestanding PMMA films. They reveal that PMMA films with one-side capped by SiO_*x*_ (gray circles) exhibit diminished relaxation strength for the fast dynamics compared with uncapped films (black circles). As shown in the inset of Figure 6d, the *f*_1_ value for one-side SiO_*x*_-capped films (the gray bar) is half that of uncapped films (the black bar), in agreement with the expectation that the fast dynamics at the PMMA–SiO_*x*_ interface are suppressed. This finding provides direct and compelling evidence that the observed long-range fast relaxation in freestanding PMMA films originates from the propagation of free surface effects into the film.

### 3.3 Investigation of the Near-Surface Dynamics by CG-MD Simulations

In a previous study,^17^ we observed a similar long-range mobility enhancement in PS. We showed that the energy of surface shear excitation modes in the films is on the order of *k*_B_ *T*, which supports spontaneous shear excitations to be a possible driver for this long-range enhancement. To explore this idea further, we employed CG-MD simulation to examine molecular motions near the surface of these polymers. The measured *T*_g_ in our model is 401 K for PMMA and is 371 K for PS (Figure S2c). These values are similar to the values obtained in a previous simulation^42^ and comparable with the values measured using ellipsometry.^55,56^ To assess MD at *T* < *T*_g_, we utilized the time–temperature superposition principle,^57,58^ which suggests that the short-term molecular behavior at high *T* is comparable with that of molecules at much lower *T* after extended aging. Based on this principle, we performed CG-MD simulations for PMMA at 1.35 *T*_g_. After achieving equilibrium, we computed the velocity field by dividing the simulation box into a 50 × 50 × 20 lattice with an average of ∼11 atoms per lattice (*N*). The average velocity (***v* ®**) of each lattice was calculated using the following formula:

5 $$\mathrm{v̅}\left(t\right)=\frac{1}{N\Delta t}\sum_{i=1}^{N}\left[r_{i}\left(t+\Delta t\right)-r_{i}\left(t\right)\right]$$

where ***r***_*i*_(*t*) is the position vector of the atom *i* at time *t* and Δ*t* equals 0.8 ns. The velocity field exhibits significant fluctuations over time. To visualize the average velocity field near the free surface, we capture a snapshot of this field in a *xy* cross-section at 1 nm below the top boundary and present it in Figure 7a,b. Each lattice is marked with a vector representing the component of ***v* ®** projected onto the *xy* plane and colored according to the magnitude of ***v* ®**.

**Figure 7:**
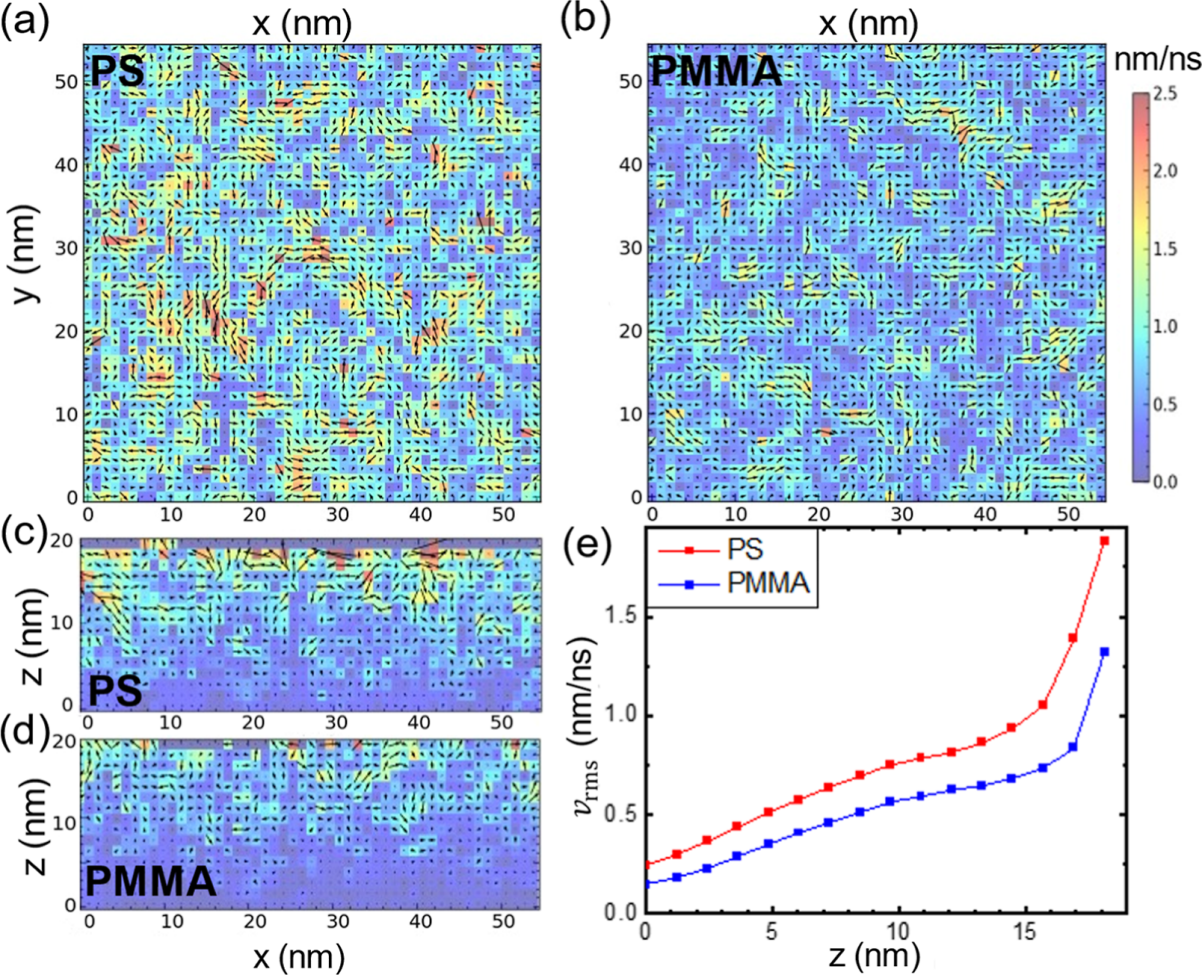
Coarse-grain MD simulations of the mobile surface layers in PS and PMMA films at 1.35 *T*_g_. (a,b) The *xy* cross-section of the average velocity field for PS (a) and PMMA (b) at *t* = 10 ns. (c,d) The *xz* cross-section of the average velocity field for PS (c) and PMMA (d) at *t* = 10 ns. The regions colored with dark gray at the top first layers in (c), where the surface is mostly devoid of atoms, are not used for subsequent analysis. For (a–d), atoms are colored according to the magnitude of the average velocity. The color scale shown in (b) applies to the data in (a,c,d) as well. (e) Root-mean-square velocity as a function of depth, *v*_rms_(*z*), for PS and PMMA. The free surface is located at *z* = 18 nm.

Figure 7c,d displays the corresponding average velocity field of the simulation box for PS and PMMA in the *xz* cross-section at *y* = 0 and at the end of the simulation. Significantly more atoms with enhanced mobility are observed near the top layer of the free surface; however, this enhancement gradually diminishes with depth beneath, persisting over distances exceeding ∼10 nm. These ${\mathrm{v̅}}_{xz}$ fields suggest that there could be coherent motions emulating from the free surface that continue to propagate inward along *z*. To quantify this trend, Figure 7e presents the distribution of the root-mean-square velocity, *v*_rms_(*z*) defined as

$$v_{rms}\left(z,t\right)=\sqrt{\frac{1}{N_{z}}\sum_{i=1}^{N_{z}}{\left|{\mathrm{v̅}}_{i}\left(t\right)\right|}^{2}}$$

where *i* denotes the lattice point, the summation is taken over lattices with the same *z*-coordinate, and *N*_*z*_ (equals 2500) is the number of lattices at the same *z*. As seen, *v*_rms_ decreases rapidly from the free surface and then declines at a relatively constant, slower rate after passing through a turning point. The rapid decrease in *v*_rms_ within the initial few nanometers of the free surface in both PMMA and PS is attributed to the thickness of the mobile surface nanolayer, which is larger for PS than for PMMA, consistent with experimental findings (Figure 2b). Furthermore, *v*_rms_ for PS is consistently larger than those for PMMA at each *z*, suggesting a longer propagation length for PS. The former observation is qualitatively consistent with the results shown in Figure 2b and the experimental findings of Zhang et al.^59^ The generally higher *v*_rms_ values align with the more significant *T*_g_ reductions found in freestanding PS films compared to freestanding PMMA films.^60^

Notably, the *v*_rms_(*z*) profiles in Figure 7e are consistent with our expectations for shear excitation modes. To further elucidate the range of these profiles and the molecular motions, we utilized a slender simulation setup, as shown in Figure 8 (left), with dimensions *L*_*x*_ = *L*_*y*_ = 9 nm and *L*_*z*_ = 250 nm. The middle and right plots in Figure 8 show the true (unwrapped) positions after long-term (200 ns) CG-MD simulations for PMMA and PS, respectively. The long-term plots clearly indicate that atoms near the surface exhibit significantly larger displacements compared to those located deeper within the films, revealing a gradual decline in the average parallel displacements from the surface to the bulk. Furthermore, the overall skewing of PS atoms in the *xy* plane is more pronounced than that of PMMA. We further observe that this skewing occurs within approximately 150 nm of the PMMA surface, similar to the *h*_t_ value found in the experiment. For PS, the skewing persists throughout the full 250 nm thickness of the simulation box, with no indication of termination, in agreement with the *h*_t_ value of approximately 1 to 3 μm for PS.^17^ While the agreement between experimental and simulated values of *h*_t_ for PMMA is impressive, it is important to highlight that the two studies were conducted at very different temperatures: the experiment at 0.86 *T*_g_ (approximately 333 K) and the simulation at 1.35*T*_g_. Nevertheless, previous studies have shown that variations in the propagation distance of enhanced mobility with temperature are gradual and typically remain well below an order of magnitude.^17,61^ Therefore, the good consistency between simulation and experimental findings regarding the magnitude of the *h*_t_ values for PS and PMMA, as well as their relative sizes, provides strong support for the simulation result. The simulation’s finding that the displacement field of these polymers exhibits long-range coherence (Figure 8) reinforces the idea that the propagation of long-range mobility enhancement may occur through shear excitations.

**Figure 8:**
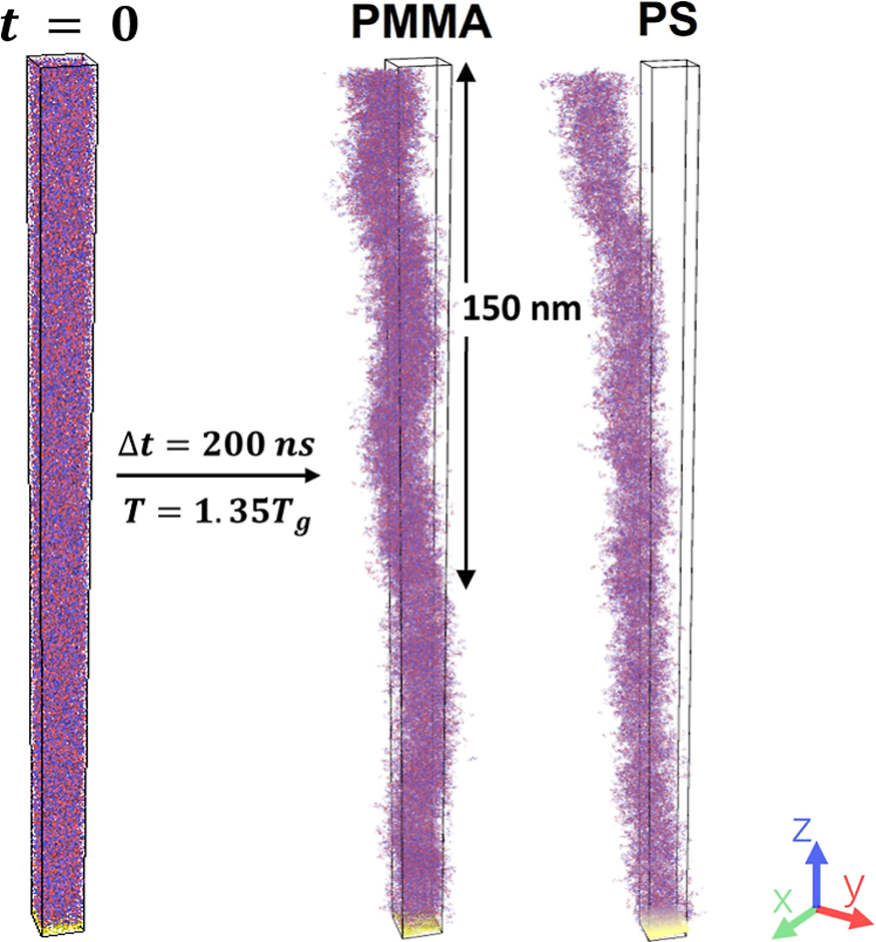
A slender simulation setup for larger-scale MD simulations of films with *h* of 250 nm (left). The long-term unwrapped position showing the long-range displacement distribution in PMMA (middle) and PS (right) films.

## 4 Conclusion

We have experimentally confirmed the presence of a mobile surface bilayer in PMMA films, comprising a surface nanolayer near the outermost region and a long-range sublayer extending ∼150 nm below. In this nanoscale outer region, the free surface effect enhances local segmental motions, while segments beneath this region exhibit long-range collective motion. Notably, the mobile surface bilayer observed in PMMA films is consistent with our previous findings in PS films, which, however, exhibit a longer, microscale sublayer.

Through CG-MD simulations of PS and PMMA films above the glass transition temperature (*T*_g_), we elucidate the mechanism driving the long-range fast dynamics. Our findings indicate that the simulated propagation length scale of enhanced mobility in PMMA films is shorter than that in PS films, in keeping with experimental observations. Furthermore, the dynamics exhibit long-range coherence, aligned with shear excitations originating from the free surface. We attribute these long-range dynamics to the collective motion of near-surface molecules, which activate subsurface molecules and generate net planar displacements through shear excitation. Future studies should investigate a broader range of glassy polymers to assess the universality of the shear excitation mode and clarify the relationship between its propagation distance and the chemical specificities of the polymer, as empirically demonstrated by PS and PMMA.

## Supplementary Material



## Notes

Heaviside-step model of film relaxation time (τ), determination of the *T*_g_ for the coarse-grain models of PS and PMMA, data justifying the friction coefficient employed in the simulation setup shown in Figure 1b, and a table of maximum modulus (*E*_max_) for PMMA films with varying thicknesses at room temperature (PDF)
