# Supplementary material for: Exploring Long-Range Surface-Induced Mobility Enhancement in Poly(methyl methacrylate)
Source: Macromolecules. 2025 Sep 18;58(19):10937–47. doi: 10.1021/acs.macromol.5c01939 (PMC12530054; doi:10.1021/acs.macromol.5c01939)
Supplement: Supplementary file 1 [file ma5c01939_si_001.pdf]

Supporting Information

Exploring long-range surface-induced mobility enhancement in poly(methyl methacrylate)

Haoran Nie[1, ‡], *Xiwen Chen*[1,2 ‡], *Zongyi Ma*[1], *Rui Zhang*[1 ,2, *], *and Ophelia K. C. Tsui*[1 ,2, *]

[1]Department of Physics, Hong Kong University of Science and Technology, 999077 Hong Kong, China.

[2]William Mong Institute of Nano Science and Technology, Hong Kong University of Science and Technology, 999077 Hong Kong, China.

Corresponding authors:

[*]O.K.C. Tsui (Email: okctsui@ust.hk)

[*]R. Zhang (Email: ruizhang@ust.hk)

[‡] These authors contribute equally.

S1. Method for calculating the mean-film relaxation time (τ) for a freestanding film with thickness *h*

A. Heaviside-step model for the relaxation time profile τ(z) in freestanding film

To analyze the relaxation time (τ) of freestanding films with h < ~200 nm, we adopted a three-layer model, where a surface layer is located at each of the two free surfaces of the film, alongside a slower layer sandwiched between them. We further assume that the dynamics within individual layer are homogeneous. The relaxation time profile τ(z) pertinent to this model is illustrated in Figure S1a, where τ_{surf} and τ_0 are the relaxation times of the surface and inner layers, respectively. This profile can be mathematically expressed by:

$$\tau(z) = \tau_{surf} + H(z - h_t^{nano}) \cdot (\tau_0 - \tau_{surf}) - H[z - (h - h_t^{nano})] \cdot (\tau_0 - \tau_{surf}) \qquad \text{(S1)}$$

where, h_t^{nano} is the thickness of the mobile surface layer at the free surface, which is found to be at the nanoscale (Figure 2b). Notably, the value of the relaxation time of inner layer τ_0 is significantly smaller than the bulk relaxation time (see their comparison, i.e., τ_0 versus τ_2 in Figure 4), indicating that long-range mobility enhancement extends over more than 100 nm from the free surface. Referring to the range of this long-range enhancement as h_t, eq S1 applies to freestanding films with thicknesses in the range $2h_t^{nano} < h \leq 2h_t$.

B. Protocol for calculating the mean-film relaxation time, $\tau(h)$, as a function of h

To evaluate the mean-film relaxation time, τ(h), for freestanding film with thickness h < ~200 nm, linear arithmetic averaging was used:

$$\tau(h) = \frac{1}{h}\int_0^h \tau(z)dz \tag{S2}$$

Figure S1b is a plot of τ(h) versus h calculated using eq S2 for the τ(z) profile shown in Figure S1a.

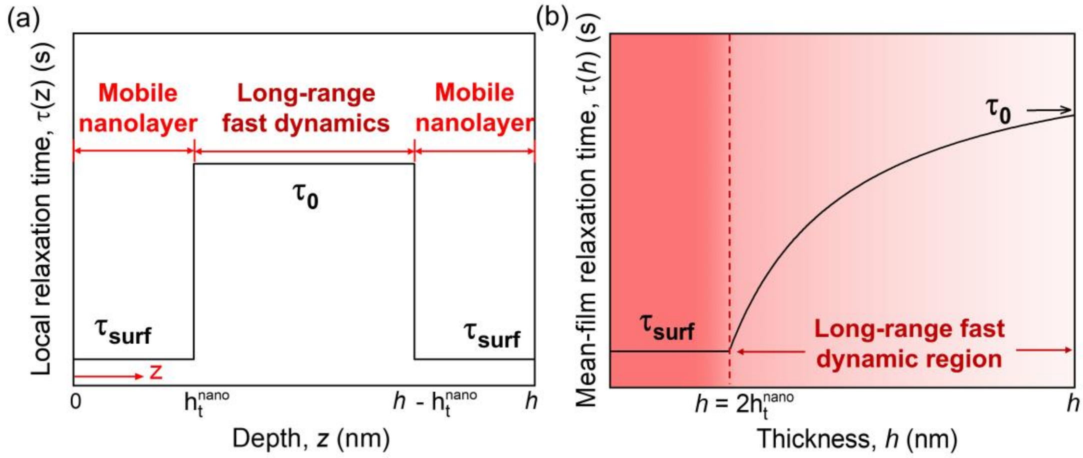

Figure S1. (a) Schematic illustration of the Heaviside-step model for the local relaxation time profile, τ(z), we adopted to analyze the mean-film relaxation time (τ(h)) of freestanding polymer films with $2h_t^{nano} < h \leq 2h_t$. (b) τ($h$), calculated using eq S2, plotted versus h.

S2. T_g determination for the CG models of PS and PMMA

A coarse-grain (CG) model of bulk polymer, devoid of any free surface, was built, as shown in Figure S2a. This model comprises 50 polymer chains, each containing 200 monomers. Periodic boundary conditions are applied to all three dimensions. The initial structure is generated using a random walk algorithm and equilibrated at 300 K using the approach described by Hsu and coworkers[1, 2]. After achieving equilibrium at 300 K, the model is heated to various higher temperatures. At each temperature, the system is equilibrated for 10 ns, during which physical quantities, such as position and velocity, are recorded every 0.4 ns for subsequent analysis.

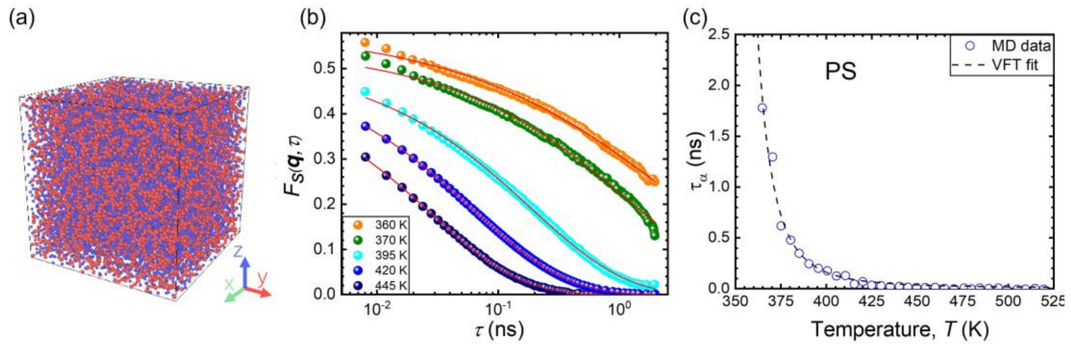

Figure S2. (a) The coarse-grain (CG) model used to determine T_g for PS and PMMA consisted of 50 chains, each containing 200 monomers. (b) Plots of intermediate scattering function $F_S(\boldsymbol{q},\tau)$ at different temperatures for PS. (c) A plot of relaxation time (τ_α) versus temperature for PS.

To determine the glass transition temperature T_g of our polymer, we first computed the self-part of the intermediate scattering function, F_S:

$$F_s(\boldsymbol{q},t) = \frac{1}{N}\sum_{j}^{N}\left\langle \exp\left[-i\boldsymbol{q}\cdot\left(\boldsymbol{r}_j(t)-\boldsymbol{r}_j(0)\right)\right]\right\rangle \qquad \text{(S3)}$$

where $\boldsymbol{r}_j$ is the position of the jth atom and $\boldsymbol{q}$ is the first peak of the structure factor $S(\boldsymbol{q})$, which is defined by

$$S(\boldsymbol{q}) = \frac{1}{N}\sum_{j=1}^{N}\sum_{k=1}^{N}\exp\left[-i\boldsymbol{q}\cdot(\boldsymbol{r}_j-\boldsymbol{r}_k)\right] \qquad \text{(S4)}$$

The magnitude of $\boldsymbol{q}$ is fixed at 15.19 nm^{-1} along x and has been found to have relatively minor effect on the results. The function F_S can be simplified to:

$$F_s(\boldsymbol{q},\tau) = \left\langle \exp\left[-i\boldsymbol{q}\cdot(\boldsymbol{r}_j(t+\tau)-\boldsymbol{r}_j(t))\right]\right\rangle_{j,t} \qquad \text{(S5)}$$

where $\langle\ldots\rangle$ denotes the ensemble average over all particles and time t. Figure S2b shows the computational results of $F_S(\boldsymbol{q},\tau)$ for PS at different T. The solid lines depict fits to the Kohlrausch-Williams-Watts (KWW) equation:

$$F_s(\boldsymbol{q},t) = C\exp\left[-\left(t/\tau_{KWW}\right)^{\beta_{KWW}}\right] \qquad \text{(S6)}$$

where C is a constant, τ_{KWW} and β_{KWW} denote the relaxation time and stretching exoponent, respectively. The structural relaxation time (τ_{α}) for each T is defined as the time at which $F_s(\boldsymbol{q}, \tau_{\alpha}) = 0.2$.

Figure S2c presents a plot of τ_α for PS at different T. The dashed line represents the best fit to the Vogel-Fucher-Tammann (VFT) equation:

$$\tau_{\alpha}(T) = \tau_0 \exp\left(\frac{\mathrm{B}}{T - T_0}\right) \quad \text{(S7)}$$

where τ_0, B, and T_0 are fit parameters. Following the approach of Hsu et al.[1-3], we define T_g as the temperature at which τ_α reaches 1 ns. Using this criterion, the T_g of PS is determined to be ~371 K (Figure S2c), while that of PMMA is ~401 K.

S3. Justification for using λ = 0.5 in the simulation setup in Figure 1b

To simulate the velocity ($\boldsymbol{v}$) near the free surface of polymer with macroscopic thickness, we apply a frictional drag force ($f = -\lambda v$) at the bottom of the simulation setup, which has dimensions $L_x = L_y = 55$ nm and $L_z = 23$ nm, as depicted in Figure 1b. To this end, the chosen value of λ should not affect the near-surface velocity of our polymers.

Figure S3a–c presents the room-mean-square velocity (v_{rms}) as a function of distance from the bottom boundary (z) of our PS and PMMA models with λ = 0.3, 0.5, and 0.7, respectively. As illustrated, the v_{rms} values at the free surface (at z = 18 nm) remain consistent across all three λ values for both polymers. This shows that a value of λ = 0.5 is appropriate for our simulation.

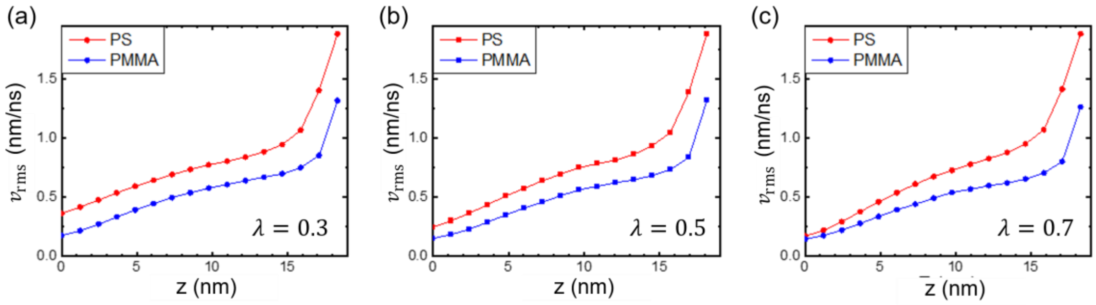

Figure S3. The v_{rms} profile obtained from the simulation setup shown in Figure 1b with λ equals (a) 0.3, (b) 0.5 and (c) 0.7.

Table S1. The high-strain rate room-temperature elastic modulus, E_{max}, of PMMA films with varying thicknesses (*h*) supported on PDMS.

***h* (nm)**	**E_{max} (GPa)**
4	2.47 ± 0.1
9	2.59 ± 0.09
12	2.67 ± 0.08
15	2.60 ± 0.10
30	2.74 ± 0.06
45	2.77 ± 0.08
68	2.88 ± 0.07
83	2.95 ± 0.05
108	2.96 ± 0.08
122	2.97 ± 0.04
200	3.01 ± 0.02

REFERENCES

1. Hsu, D. D.; Xia, W.; Arturo, S. G.; Keten, S. Systematic method for thermomechanically consistent coarse-graining: a universal model for methacrylate-based polymers. *J. Chem. Theory Comput.* **2014,** 10 (6), 2514-2527.

2. Hsu, D. D.; Xia, W.; Arturo, S. G.; Keten, S. Thermomechanically consistent and temperature transferable coarse-graining of atactic polystyrene. *Macromolecules* **2015,** 48 (9), 3057-3068.

3. Hsu, D. D.; Xia, W.; Song, J.; Keten, S. Glass-Transition and Side-Chain Dynamics in Thin Films: Explaining Dissimilar Free Surface Effects for Polystyrene vs Poly(methyl methacrylate). *ACS Macro Lett.* **2016,** 5, 481-486.
